# Psychometric Characteristics of the Brazil Mood Scale among Youth and Elite Athletes Using Two Response Time Frames

**DOI:** 10.3390/sports11120244

**Published:** 2023-12-14

**Authors:** Izabel Cristina Provenza de Miranda Rohlfs, Franco Noce, Tim J. Gabbett, Carolina Wilke, Marcelo Vido, Victoria R. Terry, Peter C. Terry

**Affiliations:** 1 School of Psychology and Wellbeing, University of Southern Queensland, Toowoomba, QLD 4350, Australia; peter.terry@unisq.edu.au; 2 Unified Center for the Identification and Development of Performance Athletes (CUIDAR), Clube de Regatas do Flamengo, Rio de Janeiro 22430-041, Brazil; 3 School of Physical Education, Physiotherapy and Occupational Therapy, Federal University of Minas Gerais, Belo Horizonte 31270-901, Brazil; fnoce@hotmail.com (F.N.); carolina.wilke@stmarys.ac.uk (C.W.); 4 Centre for Health Research, University of Southern Queensland, Ipswich, QLD 4305, Australia; tim.gabbett@unisq.edu.au (T.J.G.); victoria.terry@unisq.edu.au (V.R.T.); 5 Gabbett Performance Solutions, Brisbane, QLD 4000, Australia; 6 Health Innovation and Transformation Centre, Federation University, Ballarat, VIC 3350, Australia; 7 Faculty of Sport, Technology and Health Sciences, St. Mary’s University, London TW1 4SX, UK; 8 Executive Board of Olympic Sports, Clube de Regatas do Flamengo, Rio de Janeiro 22430-041, Brazil; marcelo.vido@flamengo.com.br; 9 School of Nursing and Midwifery, University of Southern Queensland, Toowoomba, QLD 4350, Australia

**Keywords:** Brazil, wellbeing, mood, emotion, performance, elite sport, youth sport

## Abstract

Regular assessment of the mood construct as an indicator of psychological wellbeing is used in Brazil to screen athletes for risk of mental health issues. The present study tested the psychometric characteristics of the Brazil Mood Scale (BRAMS) using both “right now” and “past week” response time frames and investigated between-group differences in mood based on athletes’ sex, age, and social vulnerability. Participants were 898 athletes (511 male, 387 female, age range: 12–44 years) from eight sports. The factorial validity of the BRAMS was supported using both response time frames independently and in a multi-sample analysis. Subscale reliability was supported for both time frames. Fatigue, depression, and tension scores were higher using the “past week” time frame than the “right now” time frame. Males reported higher vigor scores than females, and younger participants (<18 years) reported lower scores for anger and depression than older participants (18+ years). No significant differences in mood (*p* > 0.05) were found between participants identified as socially vulnerable and those who were not socially vulnerable. Findings supported the psychometric integrity of the BRAMS and its use as a screening measure for psychological wellbeing among youth and elite athletes in Brazil.

## 1. Introduction

Elite sports organizations increasingly demonstrate a commitment to safeguarding the psychological wellbeing of athletes while also striving to maximize performance [1,2]. As part of this commitment, it is commonplace for sports organizations to use some form of psychological indicator to screen for wellbeing and risk of mental health issues. Regular mood assessments have been shown over several decades to have predictive utility for both psychological wellbeing [3,4] and performance outcomes [5,6]. The mood construct is defined as “a set of feelings, ephemeral in nature, varying in intensity and duration, and usually involving more than one emotion” (p. 17) [7]. Moods have a valence dimension varying from positive (e.g., happy) to negative (e.g., depressed) and an arousal dimension varying from activation (e.g., alert) to deactivation (e.g., tired) [8]. 

Mood profiles are typically used to compare individual scores to normative scores, to assess deviations from a typical mood, as a way of screening for psychological wellbeing and risk of mental health issues [9]. Mood profiles have been shown to be effective in identifying individual athletes experiencing significant emotional difficulties, particularly when used in combination with data gathered by other sports science professionals [10]. Most psychology measures were published originally in English, presenting a challenge for those who work in other languages. Until 2006, there was no validated version of the Brunel Mood Scale (BRUMS) [11,12], a derivative of the Profile of Mood States (POMS) [13], that was suitable for use in a Brazilian context. Rohlfs and colleagues [14] addressed this gap in the literature, validating the Brazil Mood Scale (BRAMS), which represented an important precursor for further investigations in Brazil. Since its development, more than 400 published studies have used the BRAMS in, for example, sports contexts related to athlete wellbeing [15,16,17,18] and performance [19,20,21,22], and in non-sport environments related to psychological responses to illness [23,24,25] and rehabilitation [26,27]. 

### 1.1. Influence of Response Time Frame on Mood Assessment

When conducting mood research or assessing mood for applied purposes, it is important to consider how the response time frame influences the assessment of mood. It has been demonstrated that mood scores tend to vary according to whether respondents are asked to report how they feel “right now” or how they have felt “over the past week including today” [13,28]. As an example, mood scores of schoolchildren based on the “past week” time frame were significantly higher than the average scores derived from multiple “right now” assessments covering the same period; and recall of “past week” mood was highly correlated with the mood at the time of recall [28]. Hence, it is likely that respondents who are, for example, angry at the time of assessment will more readily recall incidents of anger over the past week [29]. 

An important step in testing the impact of different response time frames on the psychometric characteristics of a measure, is to evaluate whether the measurement model remains invariant across response time frames. Without that step, the mood scores of individuals or groups may not be supported by the underlying measurement structure. The BRAMS was validated for the Brazilian population to allow a quick assessment of mood states among populations of adults and adolescents, and all tables of normative data were based on the “right now” data [14], meaning that normative scores may not apply to data collected using other response time frames. In this context, the BRAMS has still to be validated using the “past week” response time frame. The developers of the original POMS, McNair et al. [13], recommended the use of the “past week” response time frame as they believed a week was long enough to capture typical emotional reactions to daily life events, yet brief enough for the assessment of acute treatment effects of psychiatric outpatients, which was their primary population of interest. They also indicated the feasibility of using other response time frames, appropriate to the purpose of a study, or applied use of the mood scale.

### 1.2. Between-Group Differences in Mood

When using standardized assessment tools such as the BRAMS, it is important to consider between-group differences because they may necessitate different tables of normative data for specific groups. Variations in mood responses have previously been identified among both athletes and the general population according to the sex and age of participants [30,31,32], which prompted consideration of these variables in the present study. Social vulnerability is another variable of particular interest within the current context. Youth athletes contending with poor housing, unstable family work conditions, and low incomes are considered high-risk candidates for mental health issues [33,34]. A mental health survey of more than 1200 residents of Rio de Janeiro’s slums, referred to as favelas, showed that over one-third experienced anxiety, depression, post-traumatic stress, or suicidal ideation [35]. Economic disadvantage is pervasive and violence commonplace in the favelas, where many athletes in the present study live. Notably, the prevalence of mental health issues in the Cruz study [35] was highest among younger people and females. Therefore, the investigation of social vulnerability as a situational variable that might influence mood scores was judged to be important. 

### 1.3. Aims and Hypotheses

The primary aim of the present study was to validate the measurement model of the BRAMS among youth and elite athletes using both “past week” and “right now” response time frames. It was hypothesized (H1) that the 24-item, 6-factor measurement model would be supported using both response time frames. It was also hypothesized (H2), based on previous evidence [13,28], that mood scores using the “past week” response time frame would tend to be higher than “right now” scores. A secondary aim was to assess between-group differences in BRAMS scores according to the sex, age, and social vulnerability of the athletes. Based on previous research [30,31,32,33,34,35], it was hypothesized that females (H3), younger athletes (H4), and socially vulnerable athletes (H5) would report more negative moods than their male, older, and socially invulnerable counterparts. The present study is necessary because the BRAMS is widely used in Brazil to assess the mood of athletes and non-athletes as an indicator of psychological wellbeing, even though the measurement model of the “past week” version is still to be validated.

## 2. Materials and Methods

### 2.1. Participants

A total of 898 athletes from a prominent multisport club in Rio de Janeiro, Brazil participated in the study. All athletes competed at least at a regional level, approximately 80% of athletes competed at a national level, and 10% at an international level. Respondents were in two groups according to the response time frame used to assess mood (i.e., “right now”, or “past week”). The “right now” group comprised 481 athletes (male = 282, female = 199) from eight sports (artistic swimming, basketball, gymnastics, judo, rowing, swimming, volleyball, and water polo) aged from 12 to 44 years (M = 17.41 ± 4.36 years). The “past week” group comprised 417 athletes (male = 229, female = 188) from the same eight sports, also aged from 12 to 44 years (M = 17.72 ± 4.54 years). A full breakdown of the participant characteristics is shown in Table 1. All athletes were members of the Unified Center for the Identification and Development of Performance Athletes (CUIDAR, which is Portuguese for “care”), a program that provides training and multidisciplinary support, encompassing medicine, nursing, physiotherapy, strength and conditioning, massage therapy, nutrition, social service, and psychology, to more than 1000 youth and elite athletes.

### 2.2. Measurement of Mood

The Brazil Mood Scale (BRAMS) [14] was used to assess mood. The BRAMS is a 24-item measure to assess the mood subscales of tensão (tension), depressão (depression), raiva (anger), vigor (vigor), fadiga (fatigue), and confusão (confusion), each of four items. One group of participants indicated how they were feeling “right now” on a 5-point Likert-type scale, where 0 = nada (not at all), 1 = um pouco (a little), 2 = moderadamente (moderately), 3 = bastante (quite a bit), and 4 = extremamente (extremely). A second group of participants indicated how they had been feeling “over the past week including today” on the same 5-point scale. Possible subscale scores have a range of 0–16, and higher scores represent higher levels of a mood dimension. The original BRUMS, of which the BRAMS is a direct translation, has demonstrated robust psychometric characteristics [11,12] and has been translated into at least 15 languages [36,37,38,39,40,41,42,43,44,45,46,47,48,49,50]. 

### 2.3. Procedure 

Data were collected over a 5-month period from April to August 2023, which encompassed a period of preparation and specific training for national and international competitions in Brazil. The BRAMS measure was presented as an online questionnaire created in Google Forms. All participants were provided with a link and instructions for completion via mobile phone under the supervision of the team coach or strength and conditioning coach assigned to their sport, all of whom had received training in the correct completion of the BRAMS. Respondents completed the BRAMS in their normal training environment. “Right now” measures were taken before or after the first training session of the week. “Past week” measures were taken at the end of the week before or after the last training session. To assess the test–retest reliability of the “right now” BRAMS, a sub-sample of 304 participants completed the BRAMS for a second time, with an intervening period of 1–6 weeks. Similarly, to assess the test–retest reliability of the “past week” BRAMS, a sub-sample of 255 participants completed the BRAMS for a second time, also with an intervening period of 1–6 weeks. All participants were informed that participation was voluntary and they provided written informed consent. Approval to conduct the study was given by the Human Research Ethics Committee of the University of Southern Queensland (#ETH2023-0046).

### 2.4. Data Analysis 

All data were collated for analysis using IBM (USA) SPSS version 29 [51] and duplicate entries from the same participants were removed except for those used for the purposes of assessing test–retest reliability. Descriptive statistics were calculated for all BRAMS subscale raw scores for both the “right now” and “past week” response time frames. Relationships among BRAMS subscale scores were quantified using Pearson correlation coefficients. To assist the interpretation of group comparisons using multivariate analysis of variance (MANOVA), BRAMS subscale scores were converted into standard scores (T-scores) by comparing raw scores to tables of normative data for adult athletes and adolescent athletes [9]. To avoid the potential for Type I errors in univariate F-tests, a Bonferroni adjustment was applied to the alpha level to account for the six dependent variables (anger, confusion, depression, fatigue, tension, vigor) by dividing *p* < 0.05 by 6, resulting in an alpha level of *p* < 0.008. The magnitude of all between-group differences was quantified using Cohen’s *d* effect sizes [52], where *d*-values of 0.20, 0.50, and 0.80 indicated small, moderate, and large effects, respectively.

The BRAMS measurement model was evaluated through confirmatory factor analysis using the IBM (USA) AMOS software [51] and several different indices were applied to assess the model. The chi-squared (χ^2^) to degrees of freedom (*df*) ratio was initially considered (where ratios of <5 and <3 represent acceptable and good-fitting models, respectively) [53]. However, the χ^2^ value tends to be significant with large samples (≥400 cases) and therefore this ratio was not used as the primary indicator of model fit [53]. Instead, two incremental fit indices were prioritized, the comparative fit index (CFI) [54] and the Tucker–Lewis index (TLI) [55], which both adjust for sample size. Values ≥ 0.90 indicated an acceptable fit and values ≥ 0.95 indicated a good fit for both the TLI and CFI. Additionally, the root mean square error of approximation (RMSEA) [56] was applied, where values ≤ 0.05 and ≤ 0.08 indicated good and acceptable fit, respectively [56]. Finally, the root mean square residual (SRMR) was used, which is a measure of the average of the standardized fitted residuals, where a value of ≤ 0.08 indicated an acceptable fitting model [53]. The samples of 481 “right now” and 421 “past week” participants met the sample size recommendations for confirmatory factor analysis [54]. 

## 3. Results

Univariate non-normality was apparent in the distributions of some BRAMS subscales (i.e., anger, confusion, depression) in both the “right now” and “past week” datasets. Negative moods often show a high proportion of low scores and a small number of high scores [11,12]. High scores on negatively valenced mood dimensions are of particular interest from an applied perspective because they suggest an elevated risk of mental health issues. Similar non-normality was found in previous validation studies involving the BRUMS [42,48,57], with adequate model fit being obtained without the need for data transformation. Further, Nevill and Lane [58] recommended that self-report data at the interval level, such as with the BRAMS, should not be transformed and hence no transformations occurred.

In the “right now” dataset, 46 multivariate outliers (*p* < 0.001) were identified using Mahalanobis distances, and another 37 multivariate outliers were identified in the “past week” dataset. All cases identified as multivariate outliers were scrutinized for response bias such as straight-line, acquiescent, or extreme responding [59,60], but none were found. Subsequently, all outliers were retained, and the final samples of 481 “right now” cases and 417 “past week” cases were included in the analyses.

Descriptive statistics, reliabilities (alpha coefficients), and intercorrelations among BRAMS subscales for both the “right now” and “past week” response time frames are shown in Table 2. Cronbach alpha coefficients for the six subscales exceeded the threshold of acceptability [61] in both samples. The negatively oriented BRAMS subscales (i.e., tension, depression, anger, fatigue, confusion) were significantly intercorrelated and either correlated inversely with vigor scores or showed no relationship. 

### 3.1. Confirmatory Factor Analysis

**Figure 1:**
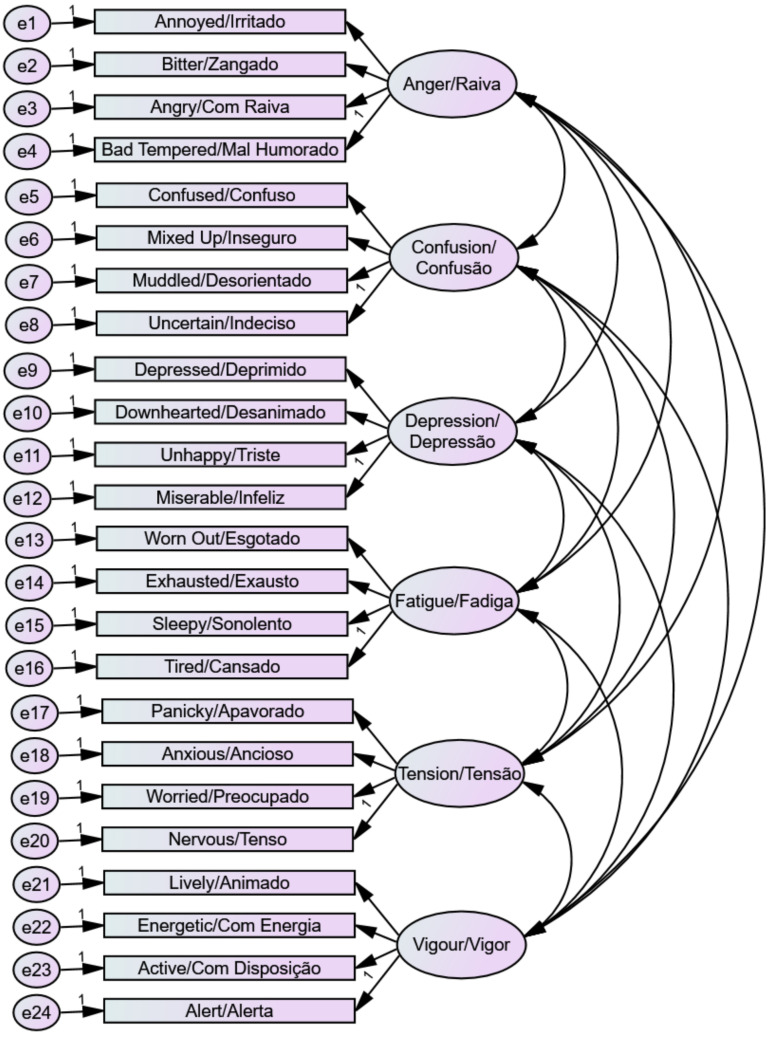
Six-factor measurement model of the Brazil Mood Scale.

The BRAMS six-factor measurement model that was evaluated using AMOS Version 29 is shown in Figure 1. Mood items and latent factors are shown in both English and Brazilian Portuguese. The results from the CFA to test measurement model adequacy of the “right now” and “past week” response time frames are in Table 3. The six-factor measurement model showed an acceptable fit with no modifications for both the “right now” and “past week” response time frames independently, and in a multi-sample CFA. Factor loadings were adequate in both samples, with 17 of the 24 items (70.8%) loading onto the corresponding factor at >0.70 and only three items (12.5%) loading at <0.60.

### 3.2. Test–Retest Reliability

For the “right now” time frame, test–retest coefficients ranged from 0.43 (anger) to 0.71 (vigor), which were very similar to those reported previously [11,12] and regarded as appropriate for a measure of transient psychological states, such as moods. For the “past week” time frame, test–retest coefficients ranged from 0.55 (depression) to 0.64 (fatigue), which were also seen as appropriate for a mood scale.

### 3.3. “Right Now” vs. “Past Week” Mood Scores

**Figure 2:**
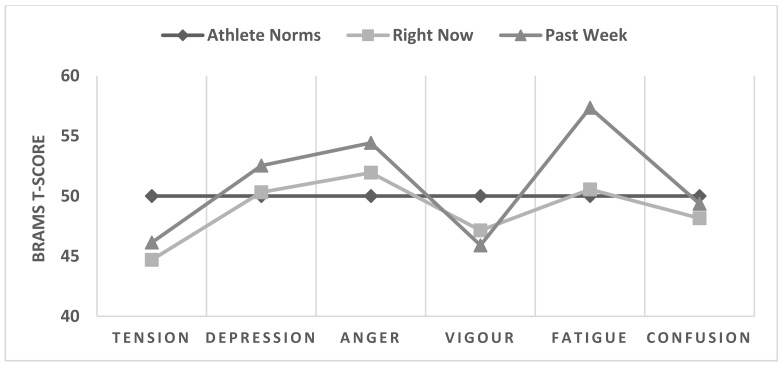
“Right now” (*n* = 481) and “past week” T-scores (*n* = 417) plotted against athlete norms [14].

Mood scores for the “right now” and “past week” response time frames were compared using MANOVA. Significant differences in mood responses between the two response time frames were identified, accounting for 8.3% of the common variance. As shown in Table 4, “past week” scores for fatigue were significantly higher than “right now” scores, with a moderate effect size. Depression and tension scores were also significantly higher when the “past week” time frame was used compared to the “right now” time frame, and in both instances, effect sizes were small. Figure 2 shows the “right now” and “past week” scores plotted against athlete norms. 

### 3.4. Between-Group Comparisons

Differences in mood responses of participants grouped by athlete sex, age group, and social vulnerability were tested using MANOVA (see Table 5). Among the “right now” dataset, significant differences in mood responses by sex were identified [Hotelling’s T = 0.143, *F* (6, 474) = 11.31, *p* < 0.001, $\eta_{p}^{2}\;$ = 0.125], which accounted for 12.5% of the shared variance. Males reported significantly higher vigor scores than females, with a moderate-to-large effect size. Comparing the two age groups [Hotelling’s T = 0.163, *F* (6, 474) = 12.88, *p* < 0.001, $\eta_{p}^{2}$ = 0.140], younger participants (<18 years) reported significantly lower scores for anger and depression than older participants (18+ years), both with moderate effects, accounting for 14.0% of the shared variance. For social vulnerability [Hotelling’s T = 0.026, *F* (6, 457) = 1.94, *p* = 0.073, $\eta_{p}^{2}$ = 0.025], the multivariate statistic was not significant, and no significant univariate differences were identified.

Similarly, among the “past week” dataset, significant differences in mood responses were also found for sex [Hotelling’s T = 0.159, *F* (6, 410) = 10.84, *p* < 0.001, $\eta_{p}^{2}$ = 0.137], accounting for 13.7% of the shared variance. Males reported significantly higher vigor scores than females, with a moderate-to-large effect size. For age group [Hotelling’s T = 0.159, *F* (6, 410) = 10.86, *p* < 0.001, $\eta_{p}^{2}$ = 0.137], younger participants (<18 years) reported significantly lower scores for anger and depression than older participants (18+ years), both with moderate effects, accounting for 13.7% of the shared variance. A marginally significant multivariate effect of social vulnerability was identified [Hotelling’s T = 0.033, *F* (6, 394) = 2.19, *p* = 0.044, $\eta_{p}^{2}$ = 0.032] accounting for 3.2% of the variance in mood scores, although no significant univariate effects were found. Two-way MANOVAs conducted to test for interaction effects among the sex, age group, and social vulnerability of the athletes showed no significant interactions for any mood subscale on either response time frame.

## 4. Discussion

The primary focus of the present investigation was to evaluate the psychometric characteristics of the BRAMS, particularly the factorial validity of the measurement model, and the internal consistency and test–retest reliability of the six mood factors. The secondary aim was to evaluate between-group differences in mood scores according to the sex, age, and social vulnerability of athletes.

As hypothesized (H1), the findings supported the factorial validity of the BRAMS and the reliability of its six factors using both the “right now” and “past week” response time frames, confirming it as a psychometrically sound scale for use in Brazilian sporting contexts. The BRAMS has been used widely in Brazil and the current validation of the “past week” version of the scale extends research opportunities to situations where retrospective assessment of mood over a 7-day period is preferred to a more immediate assessment of present mood using the “right now” response time frame. Also as hypothesized (H2), the comparison of mood scores derived from the “right now” and “past week” response time frames identified significant differences on three of the BRAMS subscales (fatigue, depression, tension), with higher scores reported for the “past week” time frame in each instance. Effect sizes were moderate for fatigue and very small for other subscales. These results mirror a previous comparison of “right now” and “past week” mood scores among schoolchildren [30], which showed moderate effects for fatigue and small effects for other subscales, with the “past week” time frame producing higher scores on all subscales. Those practitioners who use the BRAMS in applied settings should maintain awareness that using the “past week” time frame may result in higher mood scores than using the “right now” time frame as a measurement artifact rather than a reflection of a substantive change in mood. 

Significant differences in mood scores according to the sex of the athletes in the present study were restricted to the vigor subscale. Male athletes reported significantly higher scores than female athletes using both response timeframes, with moderate effect sizes in each instance. As hypothesized (H3), female athletes reported higher scores than male athletes on negative subscales for both response time frames, although the differences were non-significant and effect sizes were very small to small. These differences, although lesser in magnitude, are generally consistent with previous research, which often finds more positive moods reported by males than females among both athletes and non-athletes. Such differences are typically explained through a combination of biological [62], neurological [63], and psychosocial factors [64,65]. A previous study conducted in Brazil among 953 adolescent athletes similarly showed few differences between male and female athletes with respect to their mood scores [66].

Regarding age group differences in mood responses, counter to our hypothesis (H4), the adult (18+ years) group reported significantly higher scores than the youth (<18 years) group for anger and depression using either response time frame, showing moderate effect sizes in each case. The adult group also reported significantly higher fatigue scores than the youth group using the “past week” response time frame. These results are inconsistent with previous research findings, which have shown reported mood to be more positive among older age groups [9,32,67]. Age group differences have typically been explained (a) by the tendency of younger people to use ineffective mood regulation strategies, such as rumination, avoidance, and suppression and (b) for people to develop more effective mood regulation strategies as they get older [68]. A recent analysis of data from 29 countries involving 156,331 respondents [69] identified that the first onset of mental health disorders peaks at around 15 years of age, with the median age of onset being 19 for males and 20 for females. Hence, regular screening for mental ill-health risk among the cohort of participants involved in the present study is especially germane. 

The present results showed no significant differences in mood scores using either response time frame between athletes identified as vulnerable due to low socio-economic status and those identified as not vulnerable. This finding runs counter to hypothesis (H5), which was based on the results of a survey showing a high prevalence of mental health issues among residents of Rio de Janeiro’s favelas, where many of the athletes in the present study lived [35]. Social support is regarded as a critical factor in building physical and psychological resilience [70,71,72], and the fact that mood scores did not differ between vulnerable and non-vulnerable athletes suggests a potential protective effect of the CUIDAR program. Not only were vulnerable athletes exposed to medical and health professional support when they joined the program, often for the first time in their lives, but they also received considerable emotional and psychological support from teammates, coaches, and health professionals.

Some inherent limitations of the present study are acknowledged. Firstly, the convergent and divergent validities of the BRAMS were not assessed due to participants not having completed any concurrent measures. This was because the study was conducted in a real-world setting, which precluded the opportunity to add additional concurrent measures. It should be noted, however, that the concurrent validity of the BRAMS was established previously in the original validation study [14]. Second, given the first author’s position as manager of CUIDAR and to avoid the potential for researcher bias, the responsibility for data collection was allocated to various members of the CUIDAR support team. Although all those responsible for data collection were trained in the use of the BRAMS, the impact of data being collected by different individuals is not known and may be seen as a limitation. 

The present study is the first investigation to evaluate the measurement model of the Brunel Mood Scale in any language using the “past week” response time frame. As such, the results provide a valuable addition to the literature on the measurement of mood, particularly among sporting populations. The findings support the use of the “past week” BRAMS from a psychometric perspective. Regarding future research, the BRAMS is a suitable measure with which to investigate Brazilian populations for the prevalence of the six mood profile clusters identified in the literature, namely, the iceberg, inverse iceberg, inverse Everest, surface, submerged, and shark-fin profiles [32,66,73]. Investigating how specific mood profile clusters are related to performance and risk of mental health issues among Brazilian athletes would be another valuable future research initiative. It would be particularly worthwhile to test the predictive validity of mood profile clusters in Brazilian populations, for example, by assessing the proportion of individuals reporting the most negative mood profiles (i.e., inverse Everest, inverse iceberg, and shark fin profiles) who subsequently experience injury, overtraining, and/or mental and physical ill-health [74]. 

Evidence of the psychometric integrity of the BRAMS provides assurance of measurement validity to those using the scale to assess psychological wellbeing. In the present study, almost the full range of possible scores (range = 0–16) was reported by participants for most subscales, indicating that some athletes reported extremely negative moods, reflecting a heightened risk of psychopathology. All such individuals would be candidates for follow-up assessment by a clinically trained health professional, and it is therefore encouraging to note such follow-up is an inherent feature of the CUIDAR program, which employs a mental health specialist. The relatively small mood differences between male and female athletes and between older and younger athletes within the current sample appear to obviate the need for separate tables of normative data, and users of the BRAMS should continue to use existing norms [14].

## Figures and Tables

**Table 1:** Demographic and situational distribution of the sample (*n* = 898).

Source	Group	Right Now		Past Week	
		*n*	%	*n*	%
Sex	Male	282	58.6	229	54.9
	Female	199	41.4	188	45.1
Age Group	12–17 years	303	63.0	252	60.4
	18+ years	178	37.0	165	39.6
Social Vulnerability	Vulnerable	258	55.6	232	57.9
	Not vulnerable	206	44.4	169	42.1
Sport	Artistic Swimming	27	5.6	24	5.8
	Basketball	55	11.4	22	5.3
	Gymnastics	10	2.1	10	2.4
	Judo	40	8.3	35	8.4
	Rowing	104	21.6	98	23.5
	Swimming	75	15.6	70	16.8
	Volleyball	93	19.3	83	19.9
	Water Polo	77	16.0	75	18.0
Total	All	481	100.0	417	100.0

Note. Social vulnerability status was unknown for 33 participants.

**Table 2:** Descriptives, reliabilities, and intercorrelations for “right now” (*n* = 481) and “past week” (*n* = 417) response time frames.

Time Frame	Subscale	*M*	*SD*	Range	T-Score	α	2	3	4	5	6
Right now	1 Anger	1.29	2.46	0–14	45–137	0.87	0.56 *	0.69 *	0.50 *	0.54 *	−0.11
	2 Confusion	1.32	2.20	0–13	42–115	0.79		0.65 *	0.46 *	0.67 *	−0.05
	3 Depression	1.05	2.00	0–14	45–120	0.80			0.51 *	0.58 *	−0.22 *
	4 Fatigue	3.22	3.12	0–16	40–93	0.83				0.47 *	−0.27 *
	5 Tension	2.13	2.42	0–12	37–76	0.72					0.04
	6 Vigor	7.39	3.52	0–16	29–70	0.80					
Past week	1 Anger	1.74	2.89	0–16	45–150	0.90	0.58 *	0.71 *	0.36 *	0.58 *	−0.05
	2 Confusion	1.69	2.43	0–15	42–102	0.79		0.65 *	0.37 *	0.69 *	0.02
	3 Depression	1.49	2.57	0–15	45–139	0.87			0.37 *	0.54 *	−0.20 *
	4 Fatigue	5.26	3.83	0–16	40–93	0.85				0.35 *	−0.26 *
	5 Tension	2.63	2.76	0–14	37–80	0.75					0.12
	6 Vigor	6.89	3.48	0–16	29–67	0.78					

Note: * *p* < 0.001.

**Table 3:** Model testing of the BRAMS using “right now” and “past week” response time frames.

Group	*n*	*x^2^*	*df*	*x^2^/df*	CFI	TLI	RMSEA	SRMR
Right now 6-factor model	481	733.48 *	237	3.09	0.916	0.902	0.066	0.063
Past week 6-factor model	417	617.78 *	237	2.61	0.932	0.921	0.063	0.067
Multisample (right now/past week)	898	1360.47 *	474	2.87	0.924	0.912	0.046	0.052

Note: CFI = comparative fix index, TLI = Tucker–Lewis index, RMSEA = root mean square error of approximation, SRMR = standardized root mean square residual, * *p* < 0.001.

**Table 4:** MANOVA of BRAMS T-scores by response time frame.

Subscale	Right Now (*n* = 481)		Past Week (*n* = 417)		*F*	d
	*M*	*SD*	*M*	*SD*		
Anger	51.94	14.13	54.42	16.51	5.90	0.16
Confusion	48.15	10.11	49.35	9.69	3.26	0.12
Depression	50.31	10.54	52.53	13.40	7.74 *	0.19
Fatigue	50.55	10.43	57.34	12.76	76.97 ^†^	0.56
Tension	44.69	7.34	46.13	8.34	7.59 *	0.18
Vigor	47.14	8.62	45.90	8.50	4.68	0.14

Note: Hotelling’s T = 0.091, *F* (6, 891) = 13.48 ^†^, $\eta_{p}^{2}\;$ = 0.083. ^†^ *p* < 0.001, * *p* < 0.008.

**Table 5:** MANOVA of BRAMS “right now” and “past week” T-scores by sex, age group, and social vulnerability.

**Right Now (*n* = 481)**						
Subscale	Male (*n* = 282)		Female (*n* = 199)		*F*	d
	*M*	*SD*	*M*	*SD*		
Anger	51.22	13.43	52.95	15.04	1.75	0.12
Confusion	47.54	9.53	49.01	10.84	2.48	0.15
Depression	49.58	10.27	51.34	10.86	3.27	0.17
Fatigue	49.52	9.40	52.01	11.62	6.67	0.24
Tension	43.95	6.80	45.73	7.95	6.93	0.24
Vigor	49.45	8.02	43.88	8.39	54.09 ^†^	0.65
Subscale	U-18 year. (*n* = 303)		18+ year. (*n* = 178)		*F*	d
	*M*	*SD*	*M*	*SD*		
Anger	49.33	8.90	56.38	0.12	29.59 ^†^	0.50
Confusion	48.33	10.76	47.83	0.15	0.28	0.05
Depression	48.31	6.95	53.71	0.17	31.30 ^†^	0.51
Fatigue	49.64	9.42	52.10	0.24	6.26	0.04
Tension	44.95	7.31	44.24	0.24	1.04	0.10
Vigor	47.32	8.56	46.85	0.65	0.33	0.05
Subscale	Vulnerable (*n* = 258)		Not vulnerable (*n* = 206)		*F*	d
	*M*	*SD*	*M*	*SD*		
Anger	52.54	15.52	51.45	12.66	0.67	0.08
Confusion	48.39	11.73	47.88	7.84	0.29	0.05
Depression	51.00	12.01	49.43	8.13	2.60	0.15
Fatigue	50.73	11.53	50.59	9.21	0.02	0.01
Tension	44.31	7.52	45.32	7.26	2.11	0.14
Vigor	46.66	8.83	47.76	8.40	1.88	0.13
	**Past Week (*n* = 417)**					
Subscale	Male (*n* = 229)		Female (*n* = 188)		*F*	d
	*M*	*SD*	*M*	*SD*		
Anger	52.66	15.11	56.56	17.88	5.83	0.24
Confusion	48.51	9.83	50.37	9.44	3.83	0.19
Depression	51.29	13.10	54.05	13.64	4.43	0.21
Fatigue	56.37	12.29	58.53	13.24	2.96	0.17
Tension	45.34	8.26	47.09	8.35	4.61	0.21
Vigor	48.35	8.62	42.92	7.34	46.81 ^†^	0.64
Subscale	U-18 yr. (*n* = 252)		18+ yr. (*n* = 165)		*F*	d
	*M*	*SD*	*M*	*SD*		
Anger	51.04	10.98	59.58	21.50	28.43 ^†^	0.52
Confusion	48.97	8.36	49.92	11.44	0.96	0.10
Depression	49.73	9.26	56.81	17.15	29.74 ^†^	0.53
Fatigue	55.80	12.28	59.70	13.15	9.53 *	0.31
Tension	46.27	8.05	45.92	8.77	0.18	0.04
Vigor	46.26	8.52	45.36	8.47	1.13	0.11
Subscale	Vulnerable (*n* = 232)		Not vulnerable (*n* = 169)		*F*	d
	*M*	*SD*	*M*	*SD*		
Anger	54.31	15.92	55.09	17.84	0.22	0.05
Confusion	49.00	9.80	49.89	9.80	0.80	0.09
Depression	52.94	12.86	52.40	14.57	0.15	0.04
Fatigue	56.97	12.84	58.47	12.53	1.36	0.12
Tension	45.24	7.76	47.46	9.15	6.86	0.26
Vigor	45.17	8.89	46.64	7.84	2.98	0.17

Note: ^†^ *p* < 0.001, * *p* < 0.008. Social vulnerability status was unknown for 33 participants.

## Data Availability

Restrictions apply to the availability of these data. Data were obtained from Clube de Regatas do Flamengo, Rio de Janeiro, Brazil and are available from the corresponding author with the permission of Clube de Regatas do Flamengo.
